# Implementation facilitation to introduce and support emergency department-initiated buprenorphine for opioid use disorder in high need, low resource settings: protocol for multi-site implementation-feasibility study

**DOI:** 10.1186/s13722-021-00224-y

**Published:** 2021-03-09

**Authors:** Ryan P. McCormack, John Rotrosen, Phoebe Gauthier, Gail D’Onofrio, David A. Fiellin, Lisa A. Marsch, Patricia Novo, David Liu, E. Jennifer Edelman, Sarah Farkas, Abigail G. Matthews, Caroline Mulatya, Dagmar Salazar, Jeremy Wolff, Randolph Knight, William Goodman, Kathryn Hawk

**Affiliations:** 1 New York University Grossman School of Medicine, 227 E 30th St, Ground Floor, EM Research, New York, NY 10016 USA; 2 Geisel School of Medicine at Dartmouth College, Hanover, NH USA; 3 Department of Emergency Medicine, Yale School of Medicine, New Haven, CT USA; 4 National Institute on Drug Abuse, Rockville, MD USA; 5 Department of Internal Medicine, Yale School of Medicine, New Haven, CT USA; 6 The Emmes Company, Rockville, MD USA; 7 Springfield Hospital, Springfield, VT USA; 8 Holy Family Hospital, Methuen, MA USA

**Keywords:** Buprenorphine, Implementation science, Emergency service, Opioid use disorder

## Abstract

**Background:**

For many reasons, the emergency department (ED) is a critical venue to initiate OUD interventions. The prevailing culture of the ED has been that substance use disorders are non-emergent conditions better addressed outside the ED where resources are less constrained. This study, its rapid funding mechanism, and accelerated timeline originated out of the urgent need to learn whether ED-initiated buprenorphine (BUP) with referral for treatment of OUD is generalizable, as well as to develop strategies to facilitate its adoption across a variety of ED settings and under real-world conditions. It both complements and uses methods adapted from Project ED Health (CTN-0069), a Hybrid Type 3 implementation-effectiveness study of using Implementation Facilitation (IF) to integrate ED-initiated BUP and referral programs.

**Methods:**

ED-CONNECT (CTN 0079) was a three-site implementation study exploring the feasibility, acceptability, and impact of introducing ED-initiated BUP in rural and urban settings with high-need, limited resources, and different staffing structures. We used a multi-faceted approach to develop, introduce and iteratively refine site-specific ED clinical protocols and implementation plans for opioid use disorder (OUD) screening, ED-initiated BUP, and referral for treatment. We employed a participatory action research approach and use mixed methods incorporating data derived from abstraction of medical records and administrative data, assessments of recruited ED patient-participants, and both qualitative and quantitative inquiry involving staff from the ED and community, patients, and other stakeholders.

**Discussion:**

This study was designed to provide the necessary, time-sensitive understanding of how to identify OUD and initiate treatment with BUP in the EDs previously not providing ED-initiated BUP, in communities in which this intervention is most needed: high need, low resource settings.

*Trial registration:* The study was prospectively registered on ClinicalTrials.gov (NCT03544112) on June 01, 2018: https://clinicaltrials.gov/ct2/show/NCT03544112.

## Background

The opioid epidemic has reached a critical state, drawing widespread attention and support to address this public health crisis [1–3]. The emergency department (ED) offers a low barrier venue to initiate opioid use disorder (OUD) treatment and referral given the 24/7/365 availability for individuals with untreated OUD who often lack other sources of healthcare. In the US, ED visits associated with opioids doubled between 2004 and 2014, and visits for opioid overdose increased by approximately 30% from July 2016 through September of 2017 [4]. A recent analysis of over 17,000 ED patients who survived an opioid overdose demonstrated this population to have a 5% annual mortality rate and that only one-third received opioid agonist treatment with buprenorphine (BUP) or methadone within the year. Importantly, mortality was reduced by 59% among patients who received agonist treatment for OUD [5]. Through a landmark 3-arm randomized trial of 329 opioid dependent patients, D’Onofrio et al. demonstrated the feasibility, safety, and efficacy of initiating treatment with BUP in an urban ED [6]. Although momentum to initiate BUP for the treatment of OUD in the ED is building, adoption has been limited by the strong prevailing culture to defer initiating substance use interventions to other treatment settings [7]. This culture, combined with nearly overwhelming logistical barriers, including federal regulations restricting BUP prescribing, limited accessibility of urgent OUD referral opportunities, as well as time, space, and other resource constraints inherent to the ED setting have limited uptake of ED-initiated BUP and referral. Meanwhile, the worsening opioid epidemic is decimating communities small and large without regard to sex, race, ethnicity, or socioeconomic status [4].

## Rationale for study design

This manuscript describes the protocol, including the design considerations and methods, of a study that examined the implementation of new clinical protocols to initiate BUP for the treatment of OUD in 3 high need, low resource EDs. This study resulted from the need to quickly mobilize the resources of NIDA to translate evidence and interventions generated and tested in large, urban, resource-rich academic settings to EDs with high need for treating a growing population with OUD but limited resources. This study concept emerged to rapidly close the research to practice gap in the context of a recent randomized controlled trial finding that ED-initiated BUP is effective at improving 30-day treatment engagement [6] and high need, low resource EDs who are not equipped to initiate BUP in the ED. To overcome the aforementioned barriers to programmatic adoption, we planned to incorporate emerging technologies, specifically including a novel, injectable extended-release BUP (XR-BUP) formulation (CAM2038) whose FDA-approval was expected imminently at the time this study was initially conceptualized. Unique to CAM2038 is that it can be administered on the first day of induction to provide steady state BUP blood concentrations for seven days, enough time to arrange for a follow-up appointment with an outpatient BUP prescriber without treatment interruption. Through the robust CTN review structure, the study concept and, subsequently, the study protocol were developed and reviewed by the Protocol Review Board and Data and Safety Monitoring Board. The Emmes Company served as the Contract Research Organization (CRO), providing data and statistical support and clinical coordinating services. The study was approved by the Biomedical Research Alliance of New York (BRANY) Institutional Review Board. The study was registered on ClinicalTrials.gov (NCT03544112) on June 01, 2018: https://clinicaltrials.gov/ct2/show/NCT03544112.

## Study aims

1. To evaluate the feasibility and acceptability of implementing a clinical protocol for OUD screening and BUP treatment initiation (sublingual or extended release XR-BUP) and referral in EDs with limited resources and high need.
2. To estimate the percentage and confidence intervals of patients assessed, treated, and engaged in formal addiction treatment at Day 30.

## Study overview

The resulting study, CTN-0079: ED CONNECT: Emergency Department Connection to Care with Buprenorphine for OUD, was a three-site implementation feasibility study employing mixed methods and a participatory action research approach to: (1) develop, introduce, and iteratively refine site-specific clinical protocols for the initiation of BUP in the ED and referral for treatment of OUD and (2) evaluate both programmatic implementation and patient-level effectiveness outcomes. Prior to the study, none of the study sites, which included a community hospital, a rural critical access hospital and a large public hospital, had active ED-based BUP programs. The study builds on the aforementioned work by D’Onofrio et al. [6] and complements their subsequent, ongoing study, NIDA CTN-0069 Project ED Health, a hybrid type-3 implementation-effectiveness trial being conducted in 4 large, academic EDs that simultaneously tests both the effectiveness of ED-initiated BUP and referral as well as the Implementation Facilitation (IF) strategy employed to implement programs to initiate treatment for OUD in the ED [8].

The CTN-0079 study design and accelerated timeframe (Table 1) reflects the need to quickly implement treatment to address an urgent public health crisis. Specifically, CTN-0069 Project ED Health study methods were adapted, including IF procedures and instruments for formative evaluation and assessments, to expedite clinical and research implementation and to facilitate comparisons across these studies being conducted in markedly different ED settings [8]. Data were collected by: (i) abstracting patient data from the electronic medical record (EMR) (primary outcome, clinical program reach) (ii) conducting focus groups and qualitative interviews with approximately 60 key informants (providers/staff from the ED and community, patients, other stakeholders) (iii) administering surveys of readiness to approximately 150 ED and community OUD treatment providers and staff, (iv) enrolling a minimum of 60 ED patients who were candidates to receive ED-initiated BUP for baseline and 30-day assessments and toxicology analysis, and (v) recording qualitative field notes on implementation observations.

**Table 1:**
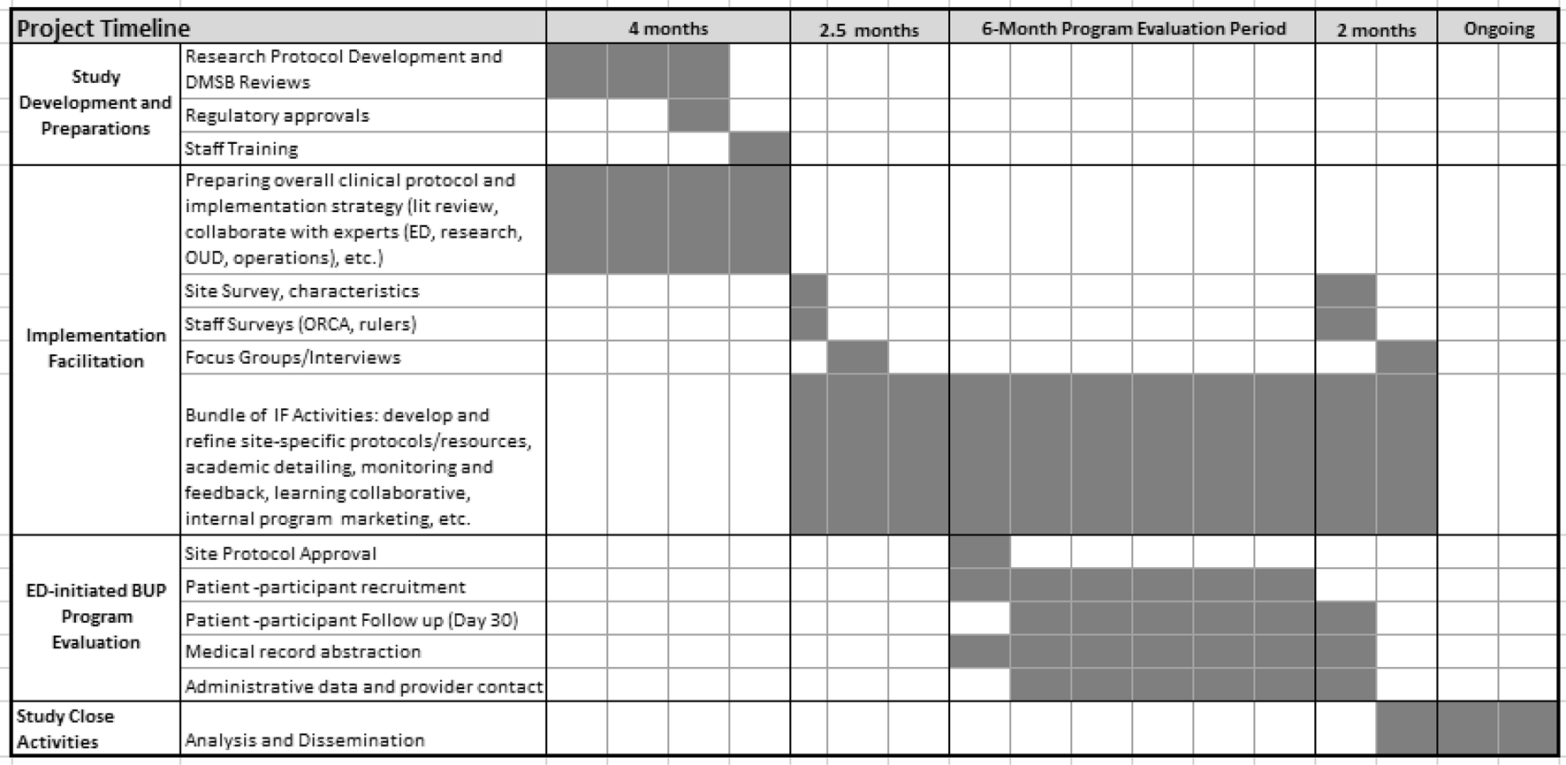
Study timeline

The study launched with the Pre-Implementation Period, during which survey data were collected and reviewed and initial site visits occurred to conduct qualitative inquiry for the formative evaluation. The six-month formal programmatic evaluation period began upon approval of each site’s clinical protocol. Investigators conducted a close-out assessment at the end of the study using the same survey and qualitative methods as were used during the formative evaluation. IF activities were initiated following the formative evaluation and continued throughout the duration of the trial to iteratively refine processes.

## Site selection

The study was conducted in three clinical EDs in hospitals with high-needs and low resources to address the opioid epidemic (as outlined in Table 2): Catholic Medical Center, Manchester NH; Valley Regional Healthcare, Claremont, NH; and Bellevue Hospital Center, New York, NY. Typically, site selection for CTN studies is performed through a formal application process with criteria to support study feasibility, generalizability, and rigor. For this study, however, settings with unique challenges to clinical implementation were selected to inform a range of implementation strategies across heterogenous EDs settings. Two of the sites were identified based on the high rates of opioid-overdose associated mortality in New Hampshire and an absence of ability to initiate BUP for the treatment of OUD in the ED. At the time, New Hampshire had the highest rate of fentanyl overdose mortality per capita in the US coupled with having among the lowest rates per capita of medication treatment providers and other treatment resources for OUD [9]. The third, Bellevue Hospital, adds a large public safety-net hospital in the midst of a hiring freeze and that, at baseline, has considerably higher patient/nurse ratios (reaching 20:1) and fewer ancillary staff than most private, community or academic EDs. Common to each site is the large proportion of economically disadvantaged or otherwise vulnerable patients served. Additionally, all sites have limited ED resources for managing a high need OUD patient population, including one ED without social work coverage and very few outpatient OUD treatment referral options. Each hospital has differing ED staffing structures; one site was changing locum tenens staffing agencies at the time of site selection, such that the ED director and all incoming providers would be newly hired. Further, all three sites used different EMR software platforms and all three announced plans to change these systems during the study period. Lastly, none offered ED-initiated BUP at the start of the study. Together, these sites enable assessment of feasibility, acceptability, sustainability and costs in heterogeneous settings including community, critical access, and municipal EDs across rural to urban population densities with varying addiction treatment and research resources.

**Table 2:** Site characteristics overview

	Valley Regional Healthcare	Catholic Medical Center	Bellevue Hospital Center
Patient volume	Low (10 K annual ED visits)	Medium–High (35 K annual ED visits)	Very High (120 K annual ED visits)
Patient need	High – High rates of OD; Fentanyl-only drug use common	High - High rates of OD; Fentanyl-only drug use common	High – High prevalence of medical-psychiatric co-morbidity and social disadvantage
Setting	Rural	Urban with suburban and rural catchment zone	Urban
Institution	Private, critical access community hospital	Private, community hospital	Municipal, Academic-Affiliated, Tertiary Care Hospital and Level 1 Trauma Center
Referral options	Low	Medium	High
ED Physician Staffing	Single coverage, non-EM trained; some locums (non-permanent staff)	Temporarily assigned, locums (non-permanent staff). Pending change to new locums agency	80 faculty members; 60 residents. Resident-driven model
ED ancillary staffing	– Social work services not available in ED	– Permanent mid-level providers	– Limited ancillary and support staff
	– No in-hospital addiction or psychiatric specialty coverage	– Limited social work support	– Extremely low nurse to patient ratios (often 1:20 in ED) and hiring freeze
			– Health coaches and volunteers screen and provide brief interventions for substance use
Space	No crowding	Significant overcrowding problem	Overcrowding is common
Unique site characteristics	Extremely limited community treatment options (none known to ED prior to study)	No ED champion. PI outside of ED. Active policy prohibiting the use of BUP in ED. Locum tenens staffing model. No existing heath system addictions care	Local expertise and partnerships exist. Understaffed and fragmented health system and referral network

## Study components

Study components were divided into (1) implementation facilitation (participatory action research approach using mixed methods), (2) evaluation of clinical protocol implementation (primary outcome and secondary process outcomes), and (3) patient-participant level outcomes (secondary outcomes of effectiveness and acceptability). See Table 3.

**Table 3:** Study components and outcomes

Study component	Outcomes		Population
Component 1—Formative evaluation and IF	Implementation outcomes	Stakeholder acceptability over time (interviews, focus groups) Stakeholder readiness/preparedness over time (ORCA, change rulers)	Community and ED key informants
Component 2—Evaluation of clinical protocol	Primary clinical outcome	Received ED-initiated BUP (proportion) (EMR abstraction)	ED patients determined to be eligible for and willing to receive ED-initiated BUP
	Secondary outcomes—Process measures and additional proportions of interest	Opioid screen completed	ED patients (adult)
		Opioid Screen positive	Screen completed
		ED-initiated BUP eligibility assessment completed	Opioid Screen positive
		ED-initiated BUP eligible	Opioid Screen positive
		ED-initiated BUP eligible and willing	ED-initiated BUP eligible
		Received a facilitated referral for treatment	ED-initiated BUP received
		Received a facilitated referral for treatment	Eligible and willing for ED-initiated BUP, but not received
Component 3—Patient-participant outcomes	Main secondary outcome	Engaged in formal addiction treatment 30 days after the index ED visit (proportion) (patient self-report with clinic confirmation)	Enrolled patient-participants who received ED-initiated BUP (secondarily, BUP non-receivers)
	Secondary outcomes—Patient treatment	Value and change from baseline for the following: Substance use (self-report via TLFB, UDS) Overdose events (self-report) Healthcare utilization (self-report Heath Services Utilization Form) Quality of life (EQ5D) Treatment satisfaction and acceptability (self-report) Initial contact with medication provider (9-day)	Enrolled patient-participants who received ED-initiated BUP (secondarily, BUP non-receivers)

## Study populations

The study populations included: (1) Key informant participants to participate in the formative evaluation and IF, (2) All ED Patients via administrative and health record data examination to assess rates of screening, assessment, eligibility determination, and (3) ED Patient-Participants who were eligible for and willing to receive ED-initiated BUP and signed written consent to participate in two research visits.

*Key informant participants* were recruited from targeted stakeholder groups to participate in the formative evaluation. All key informant participants were consenting, English-speaking adults from the below stakeholder groups who are not prisoners:

1. ED/Hospital leadership, providers, and staff across multiple disciplines (e.g., nurses, social workers, physicians, Advanced Nurse Practitioners, Physician Assistants, ED techs, pharmacists, medical directors, executive hospital leadership) at each ED site.
2. Community providers, leadership and staff involved in the provision of office based BUP, community treatment, and/or at opioid treatment programs (OTPs).
3. Other community leaders and stakeholders (e.g., EMS, fire department, police, local government leadership, community advocacy groups, etc.).
4. ED patients with OUD.

*Survey participants*: Approximately 150 members of groups 1–2 above (including those who participated in focus groups), were invited to complete structured assessments during the formative evaluation and at study close. Respondents were compensated $10 for survey completion.

*Participants contributing to qualitative data*: A purposive sample of approximately 60 individuals from groups 1–4 above across all 3 sites were recruited to participate in qualitative interviews or focus groups (based on scheduling availably) during the formative evaluation and at study close. We recruited participants that spanned disciplines and included individuals who were likely to support or resist the introduction of ED-based BUP initiation program by querying site leadership as well as focus group/interview participants themselves. Attempts were made to recruit equally across all 3 sites. Focus group participants in category 4 (ED patients with OUD) were selected based on ED patient availability and willingness during time periods of scheduled focus groups. A minority of the group 4 qualitative with OUD patient participants were selected from previously enrolled patient-participants. Each participant received a $25 gift card for participation in a focus group/interview.

*All ED patients:* Administrative and health record data for all adult patients presenting to each of the study EDs during the 6-month study program evaluation and enrollment period were examined to identify patients with OUD and potential patient-participants as well as to evaluate fidelity to clinical actions and processes.

*Patient-participants*: During the 6-month evaluation period, we intended to enroll 60 patient-participants to participate in two research visits (baseline and Day 30 post ED discharge). Given that none of the EDs had existing ED-initiated BUP programs and the limited data on which to base precision estimates, we planned to increase enrollment to our staff’s maximum potential capacity to recruit and retain (approximately 180 patients), should more robust recruitment be possible. Patient-participants were adult ED patients who were determined by ED clinical staff to be eligible for and willing to receive BUP according to criteria in site-specific clinical protocols (hereafter, referred to as “candidates” to receive ED-initiated BUP). Operationally, to be considered a BUP candidate, explicit documentation in the medical chart indicating patient interest in BUP and clinical eligibility was required unless this could be reasonably inferred by documented clinical actions (e.g., patient receives ED-initiated BUP). Patient-participants must have been willing and able to provide written informed consent, speak English sufficiently to understand study procedures, and provide two unique forms of contact, and were excluded if currently engaged in medication for OUD (MOUD) treatment or opioid-requiring pain management, a participant in a substance use intervention study, medically or psychiatrically unstable, or a prisoner. We employed a recruitment strategy to ensure patient-participant enrollment would be relatively even over time and between sites, and that the ratio of patient-participants who receive BUP to those who do not would be at least 2:1, as we anticipated that some eligible patients would not receive buprenorphine. Participants were compensated $75 upon completion of screening and baseline and $100 upon completion of the 30-day follow-up visit.

## Study component 1: implementation facilitation

Implementation Science, defined by the National Institute of Health as “the study of methods to promote the integration of research findings and evidence into healthcare policy and practice” [10] provides an organized approach and tools to fill the gap between the need and provision of ED-initiated BUP and ongoing medication treatment for OUD. We employed the IF methodology, using procedures adapted from those used in CTN-0069 Project ED Health [8], which are based on a manualized program developed by Kirchner and colleagues [11]. For this systems-level intervention, study external facilitators engage with stakeholders and identify and collaborate with local champions to conduct a formative evaluation, develop and refine clinical protocols resources, perform academic detailing and staff education, lead a learning collaborative, and facilitate performance monitoring and feedback. These elements of our IF approach are outlined in Table 4 and specified further below.

**Table 4:** Implementation facilitation roles and activities

Role	Definition
External facilitators (EF)	Study investigator content experts (McCormack, Hawk) facilitate activities (as described below) designed to promote implementation of the clinical protocol for OUD tailored to the clinic-specific needs
Local champions (LC)	LCs were ED clinical staff who help promote ED-initiated BUP with referral for treatment. LCs will serve the primary liaison between the ED and the EFs. LCs will lead implementation efforts on the ground, identify site-specific needs, and work with department and hospital leaderships to draft policies and secure approvals
ED staff and providers	All ED staff and providers were invited to participate in the Learning Collaborative, receive training/education, and provide feedback on the implementation of clinical protocol

Activity	Definition
Formative evaluation	Using mixed-methods, the research team identify evidence, context, and facilitation-related factors impacting the provision of ED-initiated BUP with referral for treatment in the community and use these data to refine and evaluate the effectiveness of the IF
Advising on ED-initiated BUP Clinical Protocol Development	Serving in an advisory and consultant capacity, EFs work with the clinical sites to develop a clinical protocol for nonmedical opioid use screening and ED-initiated BUP with facilitated referral tailored for their site. EFs will provide ongoing consultation to help monitor, support, and refine implementation
Assistance with facilitated referrals	EFs will work with LCs to identify community OUD treatment providers and create site-specific referral lists of medication treatment providers and other supportive resources for patients with OUD. EFs will also assist with identifying a practical approach to facilitating referrals
Stakeholder engagement	Stakeholder engagement took place in the form of in-person meetings at the administrative, provider, community and patient levels. Efforts at increasing engagement were informed by the focus groups and qualitative interviews and supported by the efforts of the LCs
Tailor program to site	The IF strategy were tailored to the local site as informed by the formative evaluation, involvement of the LCs, and with feedback from all ED staff and providers
Provider education and academic detailing	All ED providers were offered educational sessions on OUD and BUP training, specifically tailored to each provider’s tasks. We will address practical issues such as efficient use of the EMR for prompts, provide tools and web-based resources, and share patient monitoring strategies
Performance monitoring and feedback	We worked with ED leaders and other members of the ED staff to incorporate clinician performance related to BUP-initiation and facilitated referral into the department’s standard quality improvement and feedback practices. Sites were provided aggregate feedback on screening for nonmedical opioid use, adherence to clinical actions, eligible patients receiving BUP in the ED and referred patients’ enrollment in ongoing treatment
Learning collaborative	A Learning Collaborative was formed by inviting each of the site’s LCs and other ED stakeholders to participate in weekly conference calls to promote shared learning regarding issues promoting and hindering implementation of addiction treatment. Topics will include DATA 2000 “x-waiver” requirements, strategies for launching a new clinical initiative, existing models of ED-BUP, and BUP education, among others

*Formative evaluation*: Throughout the study timeline, we used a participatory action research [12] approach, adapted from the IF strategy in CTN-0069 Project ED Health [8], to iteratively gather information from stakeholders and key informants to inform the planning and execution of actions to refine procedures and support implementation and enhance acceptability. This was achieved by holding regular stakeholder meetings and conducting and repeating qualitative and quantitative assessments with clinical and administrative staff, patients, and other stakeholders. IF was guided by formative evaluation, an iterative process that uses these mixed methods to tailor training, support, and overall implementation of the clinical protocol to each specific site. Formative evaluation included site-specific organizational, provider, and patient factors potentially impacting uptake of provision of ED-initiated BUP [11]. Throughout the implementation period, we rigorously documented summary findings, notable observations, and preliminary themes from various sources (interviews, focus groups, stakeholder meetings, learning collaboratives, IF logs maintained by research staff at each site, monthly clinical staff meetings at each site, and other feedback), which we entered into action research matrixes. We had multiple standing calls per week with research staff and various stakeholders to review, provide feedback, and add to these data. By organizing and continuously updating data triangulated from multiple sources along with corresponding subsequent actions consistent with a Rapid Assessment Process[13], these matrixes provided the structure to rapidly synthesize preliminary data and iteratively refine clinical protocols and implementation strategies.

*PARiHS framework*: Building on the mixed-methods analysis conducted during the formative evaluation, we used the Promoting Action on Research in Health Services (PARiHS) framework to tailor IF for site-specific needs [14–17]. We characterized the facilitators and barriers identified by the key informants according to the PARiHS sub-elements of patient and clinical experience (communication, knowledgeable and empathetic providers), receptive context (resources to provide addiction treatments), and culture (value of team-based approach) identified. PARiHS was used to further explicate and design the IF, guide the ongoing formative evaluation, and revise the strategy in an iterative manner to improve implementation success. We iteratively assessed processes and received feedback from providers, patients, and other stakeholders to amend and improve the feasibility, acceptability, and uptake of ED-initiated BUP in a way that is sustainable across the different sites.

*Clinical protocols and resources development:* Our clinical protocol development strategy included developing clinical protocols containing critical clinical actions of the intervention to expected fidelity (i.e., identifying and appropriately assessing patients for treatment, initiating BUP treatment, facilitating referral, etc.) along with aspects that may be adapted by local sites to aid implementation. The Yale clinical protocol, previously tested by D’Onofrio et al., served as the base case model [4]. In partnership with multidisciplinary teams at each site, we adapted clinical practices and available information about SL-BUP and XR-BUP to site-specific clinical protocols and implementation strategies. The resulting algorithms provided guidance related to the choice of formulation, dose, timing, and other decisions, including whether home induction with SL-BUP is appropriate. The site-specific clinical protocols were refined throughout the entirety of the study to improve programmatic feasibility, acceptability, and effectiveness using the Rapid Assessment Process of collecting and synthesizing data [6]. This form of participatory action research is an intensive, team-based approach, involving rapid cycles of gathering information, planning actions, implementing changes, and collecting feedback to inform subsequent revisions. It is ideally suited for this study because it allowed us to explore and test modifications to how, when, and by whom critical clinical actions are performed across these markedly different ED settings with unmet treatment burden, and to disseminate generalizable information expeditiously to support OUD treatment where it is needed most.

*Resource development*: Similarly, serving in an advisory capacity, external facilitators worked with local champions to identify potential OUD treatment providers for ongoing treatment and draw on existing resources to support programmatic implementation. These resources were to simply and practically help providers gain competence and confidence in identifying candidates for ED-initiated BUP, performing pre-induction assessments, inducting patients onto BUP in accordance with clinical prescribing guidelines and facilitating referral for treatment. External facilitators identified existing resources and technologies that aid effective implementation by, for example, aiding to engage and train clinical staff, provide real-time clinical guidance/support, reduce stigma-related barriers, and improve clinical documentation and quality assurance monitoring.

*Education and academic detailing:* External Facilitators, who work clinically as emergency medicine physicians and have expertise in initiating BUP in the ED and formal training in academic detailing (https://www.narcad.org), conducted site visits and met with individuals and/or in small groups with clinical staff working in the ED at each study site to perform academic detailing, which involves sharing unbiased information about patient assessment and treatment with the goal of improving quality of care [11]. Clinicians who may be involved in the initiation or continuation of BUP or assisting with the referral process were offered educational sessions on OUD and BUP training, specifically tailored to each provider’s tasks. We addressed practical issues, such as efficient documentation, and offered opportunities and facilitated training, including completing the Drug Addiction Treatment Act of 2000 (DATA 2000) waiver (i.e., X-waiver) for BUP prescribing. Data from the formative and ongoing evaluation were used to amend strategies to enhance implementation.

*Learning collaborative*: A weekly Learning Collaborative was formed by inviting each of the site’s local champions, and other key stakeholders, to participate in weekly, interactive conference calls to promote shared learning regarding issues promoting and hindering implementation of addiction treatment. This call provided a dedicated time to discuss site-specific clinical updates, challenges and possible solutions for implementation of addiction services.

*Performance monitoring and feedback:* We worked with ED leaders and other members of the ED staff to incorporate clinician performance related to BUP-initiation and facilitated referral into the department’s standard continuous quality improvement and feedback practices.

## Study component 1: IF assessments and measures

*Site assessments:* Site characteristics surveys were completed by clinical directors or their designees for each site at the start and end of the study timeline to gather information describing the ED, hospital, and community treatment programs, including staff characteristics (age, sex, training, permanent/Locum Tenens, etc.), existing and potential treatment services available in the ED and community, and patient payer mix and demographics. Included in this were the number of providers who have obtained DATA 2000 waivers to prescribe BUP.

*Provider and staff quantitative assessments (surveys):* The quantitative components included anonymous web-based surveys of ED and community treatment setting providers and other staff. An introductory email and reminders containing a link to the survey was sent on behalf of their respective leadership explaining the purpose of the survey. When completing the survey, respondents consent for their information to be reported in aggregate. Baseline surveys were completed prior to IF activities and repeated at study close, including the Organizational Readiness to Change Assessment (ORCA) and change rulers which were both previously adapted to assess readiness and preparedness to provide ED-initiated BUP [18]. Based on the PARiHS framework, these measures were used to determine evidence- and context-related strengths and weaknesses in organizational and personal readiness to implement BUP and referral and to tailor the IF. Each respondent also completed a brief Individual characteristics survey that gathers information on clinical role, training, treatment of OUD and general demographic information.

*Key informant qualitative assessments: focus groups and interviews*: The qualitative components, consisting of semi-structured interviews and focus groups, provided a more in-depth understanding of feasibility and acceptability, including barriers, facilitators, and other needs to support implementation, from the perspectives of ED and community treatment setting staff as well as ED patients and community stakeholders [key informant participants]. We chose to use focus groups given their suitability for generating data from multiple perspectives regarding the organizational and individual level factors impacting complex processes when available, and used one-on-one semi-structured interviews for information gathering to allow for the broadest inclusion of perspectives when it was neither feasible nor practical to arrange a suitable focus group [19]. The interview guide used in CTN-0069 Project ED Health [8] was adapted to specifically elicit perspectives about BUP and better understand how the characteristic differences of the sites and the population served by them may influence programmatic implementation and effectiveness. All participants provided verbal informed consent to participate in a 60-min focus group/interview that was recorded and transcribed verbatim.

## Study component 2: evaluation of clinical protocol implementation (primary outcome)

Process measures related to the clinical protocol as well as reasons for non-completion and/or ineligibility were abstracted from the hospital EMR. These data allowed for measurement of clinical protocol adherence to support both overall study goals and site-specific internal continuous quality improvement efforts. This dataset provided the study primary outcome, which we define from Aim 2 as the proportion of unique patients who received ED-initiated BUP amongst patients who were determined to be eligible for and willing to receive ED-initiated BUP. Under a waiver of consent, research staff reviewed data for all patients presenting to the ED at each study site during the study enrollment period. Individual charts of those patients screening positive for nonmedical opioid use were reviewed to collect documentation of adherence to components of the clinical protocol using an electronic data capture system managed by the Data and Statistics Center at the Emmes Company. We generated proportions along the cascade of clinical actions and measure fidelity to critical and non-critical clinical actions, including but not limited to the following: screening for non-medical opioid use, assessment of OUD, assessment of opioid withdrawal, assessment of pregnancy among women, initiation of BUP, prescription for ongoing BUP, and facilitation of referral.

Rigorous research staff training was followed by weekly meetings to review cases and quality assurance monitoring. A second member of the research team independently performed medical record abstraction of a random sample of approximately 10% of the logs created (i.e., 10% of the days in the enrollment period). The second independent reviewer was masked to the information obtained by the first reviewer. Once the second review was completed and entered, the data system performed a comparison of the two logs and generated discrepancy reports. Inter-rater agreement between the two independent coordinators for the chart abstraction required for the primary outcome data was measured by generating a kappa statistic. Periodic meetings with chart abstractors and other team members (i.e., site investigators) were held to resolve discrepancies, review coding rules, and monitor performance.

## Study component 3: patient-level outcomes

We explored our secondary patient-level outcomes (engagement in ongoing treatment, drug use, overdose events, healthcare use, quality of life, acceptability, etc.) by recruiting ED patients who were candidates for ED-initiated BUP to participate in two research visits. Research staff worked rotating shifts in the ED, providing coverage on weekdays, evenings and weekends. Study staff did not approach patients (or their providers) for potential study entry until all clinical actions included in the site’s ED-initiated BUP protocol were completed (i.e., screening, treatment initiation, and referral) or after the ED visit was completed. Thus, if any actions remained incomplete and/or the research screening could not occur during the index ED visit, the study coordinator approached the patient after discharge from the ED. This decision to delay our approach was deliberated at length. Although it added considerable challenges to patient recruitment, it minimized the risk of research activities influencing the clinical processes being evaluated; therefore, it minimized potential confounding of our primary implementation outcome, which is evaluated via EMR abstraction without patient-participant enrollment. Potential study participants who presented to the ED outside of recruiting hours or could not be approached in person were contacted and screened telephonically using contact information available in the EMR. At each site, patients were able to opt-out of such communications. Remotely approached potential participants who were eligible for and interested in study participation were scheduled for written informed consent and baseline assessments to be completed in person within 7 days of their ED discharge.

*Baseline visit:* Adult ED patients who were determined to be eligible for and willing to receive BUP by ED clinicians according to site-specific clinical protocols were approached by research staff to be screened for potential study participation. Both candidates who did and did not receive BUP were enrolled to learn about the acceptability of and barriers to initiation of BUP in the ED. Before performing any study assessments, research staff requested the patient’s verbal consent to assess eligibility using an IRB-approved verbal consent script. After the patient provided verbal consent, research staff collected basic demographic information and confirmed that the candidate met all the inclusion criteria and none of the exclusion criteria using an eligibility checklist. To participate, eligible candidates provided written informed consent and signed an authorization for release of information for the purpose of confirming treatment engagement at day 30 with treatment facilities. After providing their written informed consent, enrolled patient-participants provided a urine sample for drug testing and completed coordinator-administered assessments (described below), which required approximately 30–60 min. Upon completion of the baseline visit, patient-participants were scheduled for a follow-up research visit to occur 30 days after the index ED visit.

*Follow-up research visit (Day 30)*: All patient-participants were asked to return to a research office on the hospital campus for a follow-up research visit 30 days after their index ED visit. Study coordinators used multiple forms of contact and locator information collected during enrollment to remind patient-participants of the visit; IRB-approved scripts for telephone, email, and other messaging were used for all communications to support study retention. At the follow-up visit, patient-participants provided a urine sample for drug screening and repeated the assessments completed at the baseline visit as well as additional questions related to engagement in formal addiction treatment and treatment satisfaction.

## Patient-participant measures

The study team obtained data by participant self-report, EMR abstraction, direct contact with treatment providers, and review of the Prescription Drug Monitoring Program (PDMP) database. We assessed a range of pretreatment participant characteristics derived from our team’s previous and ongoing studies, including CTN-0069 Project ED Health [8], and the Substance Abuse and Addiction Collection of the PhenX Toolkit that includes measures that are being adopted across NIDA-funded research (Table 5). Baseline assessments and patient reported measures were used to ensure that patients met eligibility criteria, that important predictor variables are assessed, and that we have a baseline for changes in patient-reported outcomes. Biologic specimens included urine samples analyzed for opiates, methadone, oxycodone, cocaine metabolite (benzoylecognine), barbiturates, methamphetamine, amphetamine, marijuana and benzodiazepines. Fentanyl was tested using the BNTX Rapid Response™ fentanyl urine strip test, with a detection level of 20 ng/ml norfentanyl (for forensic use only) [20]. Research staff confirmed participant-reported engagement in formal addiction treatment on the 30th day after the index ED by contacting the treating provider/facility reported by the participant. Additionally, for each participant, study staff reviewed the EMR and PDMP database to abstract data on treatment delivered during the index ED visit and hospitalizations, ED visits, and prescriptions for BUP and other opioid analgesics filled in the subsequent 30-day period.

**Table 5:** Schedule of research assessments for patient-participants

Assessment	Screening	Baseline	Day 30
*Eligibility and enrollment*			
Verbal consent	X		
Prisoner status assessment	X		
Demographics	X		
Eligibility summary	X		
Enrollment (inclusion/exclusion)	X		
*General*			
Written informed consent and medical release	X		
Additional demographics		X	
Locator information form		X	X
DSM-5 checklist for OUD		X	
Other substance use [21]		X	
EuroQol-5 dimensions (EQ-5D) [22]		X	X
Motivations, attitudes and expectations		X	
Study completion			X
*Health services*			
Inpatient utilization		X	X
Outpatient utilization		X	X
Health status [23–25]		X	X
Healthcare visit logistics		X	
ED visit review		X	
ED visits and hospitalizations			X
*Process outcomes*			
Engagement in treatment			X
Prescription drug monitoring			X
Treatment decision		X	
Treatment satisfaction/acceptability			X
*Opioid outcomes*			
Timeline follow-back, 7-day (TLFB) [26, 27]		X	X
Urine drug screen		X	X
Overdose events and risk factors		X	X
*Safety*			
Safety events		X	X

### Data and safety monitoring

Because this prospective study examined BUP treatment initiation and the impact of ED-initiated BUP on engagement in addiction treatment and drug-use-related outcomes, and the use of these medications is in line with community practice, safety reporting was limited to recording any opioid overdose that occurs on study, any death, and healthcare utilization including ED visits and hospitalizations. Each of the sites and communities have established practices for managing medical and psychiatric emergencies, and those established practices were followed per standard of care in each community.

An independent study monitor was designated to review all safety events for this protocol and determine if reporting to NIDA, the DSMB and/or regulatory authorities was required. Reports were generated by the data and statistics team at the Emmes Company and presented for DSMB meetings. An independent CTN DSMB examined accumulating data to assure protection of participants’ safety and assure that scientific goals were being met. The CTN DSMB is responsible for conducting periodic reviews of accumulating safety trial performance and outcome data. It determined whether there was support for continuation of the trial, or evidence that study procedures should be changed, or if the trial should be halted, for reasons relating to the safety of the study participants or inadequate trial performance (e.g., poor recruitment).

### Statistical analysis

Using a participatory action research approach [12] and mixed methods, we developed, introduced, and updated site-specific ED clinical protocols and implementation plans for OUD screening, treatment, and referral to optimize feasibility and acceptability. Barriers to- and facilitators of implementation were explored, and the impact of remediation efforts were assessed through sequential qualitative and quantitative inquiry and feedback generated through the learning collaborative. Converging provider and patient perspectives with process measures and intervention outcomes, including proportions screened, treated, and remaining engaged in treatment, provided explanation to contextualize and better understand feasibility, acceptability, and patient-level outcomes.

### Qualitative statistical analyses (component 1)

To inform iterative changes that occurred throughout implementation phase we used the Rapid Assessment Process, an intensive, team-based qualitative inquiry using triangulation, iterative data analysis, and additional data collection to quickly develop a preliminary understanding of a situation from the insider’s perspective [13]. Multiple team members debriefed after each focus group, interview and stakeholder meeting to review field notes, discuss findings and generate a summary report collectively. These summary findings were entered into the action research matrixes to inform real-time changes during the implementation period and were shared with key stakeholders. Concurrently, using the full transcripts, we began the formal qualitative analyses, understanding it would continue beyond the programmatic implementation and evaluation periods.

Qualitative outcomes are analyzed using directed content analysis [28]. The transcripts will be independently reviewed, coded and analyzed by a multi-disciplinary group. Initially transcripts will be individually reviewed line by line in entirety and coded by multiple independent team members. Following the coding of the initial set of transcripts, the qualitative research team will meet to review the initial coding scheme and a codebook will be generated by consensus, which will contain operational definitions for each code. Code generation will be iterative and the codebook subject to change until no new codes are identified. Common patterns across the dataset will be identified and will be grouped into themes. Analysis will use the PARiHS framework, which examines the interaction between three key elements of Evidence, Context and Facilitation, and including sub-elements of patient and clinical experience (communication, knowledgeable and empathetic providers), receptive context (resources to provide addiction treatments), and culture (value of team-based approach). An audit trail will be maintained. Data will be entered and organized using Atlas.ti software.

The technique of triangulation, in which the data from different types of ED and community staff and providers, including nursing, social work, administrators, physicians, physician assistants and advanced nurse practitioners are interpreted in the context of each other and patient perspectives to better understand facilitators and barriers will be used. In addition to triangulating by different sources of qualitative data, data will be interpreted in the context of other types of data available, including data abstracted from EMR and administrative databases, and quantitative data from patient-participants.

### Analysis of survey responses (component 1)

Initial and changes in readiness and preparedness scores (ORCA, readiness and preparedness rulers) as well as site characteristics were analyzed using descriptive statistics with pre-post comparisons, when appropriate.

### Analysis of primary clinical outcome (component 2)

The second aim was to estimate the percentages and confidence intervals of patients assessed, treated and engaged in treatment at day 30. We defined the primary outcome as the probability of an individual receiving ED-initiated BUP given that the individual was determined to be eligible for and willing to receive ED-initiated BUP using patient-level data abstracted from the EMR. We refer to this below as the implementation probability, $p$. To avoid difficulties with zero counts, we used Bayesian estimates for the site-level implementation probability values, assuming beta likelihood uniform prior to derive posterior moments. That is, if there were S successes (i.e., BUP initiated) and F failures (i.e., BUP not initiated) for a site, the $p$ estimate for that site would be $\alpha /(\alpha +\beta )$, with estimated variance $\alpha \beta /[{\left(\alpha +\beta \right)}^{2}\left(\alpha +\beta +1\right)]$, where $\alpha =S+1$ and $\beta =F+1$. The overall $p$ estimate would be the average of the three site-level estimates. Because the site-level $p$ estimates are independent, the variance of the overall $p$ estimate would be the sum of the site-level variances divided by 9. To construct confidence limits, we assumed the overall estimate is roughly normal in distribution, with upper and lower 95% confidence intervals given by $\pm 1.96* \surd {V}_{overall}$. An exception to this is that we did not allow confidence limits to stray outside (0,1).

*Power/precision calculations:* For our analytic plan, we assumed that 120–180 individuals (ED patients), identified evenly from 3 sites, would be available to investigate this primary outcome. However, there was little data on which to base these estimates as none of the sites had existing clinical programs to screen for or treat OUD. Simulations conducted prior to enrollment that introduced variability among sites showed that bias was not large for most parameters investigated, and confidence intervals wider than 0.4 for $p$ were not expected.

### Analysis of process measures and additional proportions of interest (component 2)

Using EMR and administrative data, we assessed fidelity to critical actions related to the program (screening, enrollment, medication administration, and navigation to formal addiction treatment, etc.) and other process measures of interest (Tables 3, 5). For each of these, descriptive statistics were performed, including proportions for categorical data and means, standard deviations, minimum, and maximum for continuous variables. We assessed differences between sites and the change in proportions over time since clinical protocol implementation.

### Analysis of patient-participant secondary outcomes (component 3)

Analysis of the most important secondary outcome, i.e., the probability of being engaged in formal addiction treatment on the 30^th^ day following the index ED visit amongst enrolled participants who received ED-initiated BUP, uses methods similar to that of the primary outcome. We expected to be able to enroll a sample of at least 42–60 patient-participants with which to assess this and other secondary outcomes (see Table 3), and actually enrolled 40 participants who received ED-initiated BUP.

Patient-reported outcomes (e.g., illicit opioid use, overdose events, healthcare use, quality of life, treatment satisfaction) were analyzed using descriptive statistics with pre-post comparisons (i.e., baseline to day 30) reported, when appropriate. We used appropriate non-parametric, parametric, and analysis of variance statistical procedures to descriptively evaluate the key characteristics of each study site and to evaluate comparability of baseline characteristics among patient cohorts enrolled at each of the study sites and overall during the study. As a non-randomized study, formal and rigorous hypothesis-testing was not carried out. Findings will be reported as noteworthy hypothesis-generating results only when their p-values are considerably smaller than 0.05.

**Fig. 1:**
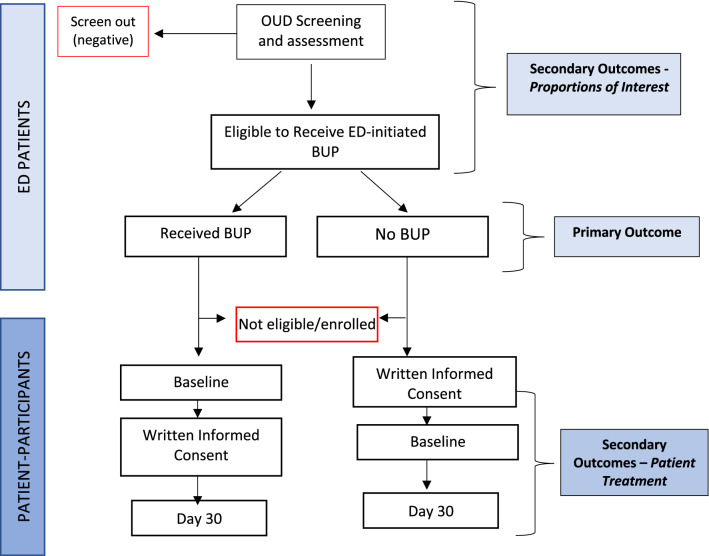
Diagram of patient-participant enrollment and outcomes

*Missing data:* The flow diagram of ED patients and patient-participants (Fig. 1) will include information on ineligibility and loss to follow-up. There was no loss to follow-up for the implementation probability primary outcome, because all necessary data is available through the EMR. With respect to the secondary Day 30 treatment engagement proportion, patient-participants lost to follow-up after receiving ED-BUP were counted as not engaged in treatment, thus contribute to both numerator and denominator instead of generating missing data.

## Discussion and conclusion

The opioid epidemic has a large and growing impact on public health and continues to decimate communities ill-equipped to provide substantive, timely intervention. As the receiving center for persons experiencing overdose, the call to action is reaching the ED. While the ED may be an ideal and underutilized venue for addressing this crisis, it is well-recognized to be an extremely challenging venue for introducing, sustaining, and studying interventions. By assembling subject matter experts and involving local stakeholders, we will translate successful elements of efficacious interventions to EDs operating in different contexts. These partnerships provide an opportunity for prompt, meaningful and sustainable dissemination with enhanced support for the intervention while it is being developed and tested in situ. This study is designed to provide the necessary, time-sensitive understanding of how to identify OUD and initiate treatment with BUP in the EDs where this intervention is most needed—which, if successfully done, should save lives, improve outcomes, and reduce costs to society.

## Data Availability

De-identified versions of datasets will be provided to the NIDA CCTN-designated parties for posting on Datashare, as well as storage and archiving. Reference: http://grants.nih.gov/grants/guide/notice-files/not98-084.html.

## Abbreviations

BRANY: Biomedical Research Alliance of New York
BUP: Buprenorphine
CRO: Contract Research Organization
CTN: Clinical Trials Network
DATA 2000: Drug Addiction Treatment Act of 2000
DSMB: Data Safety and Monitoring Board
ED: Emergency Department
EF: External facilitator
EM: Emergency medicine
EMR: Electronic medical record
EMS: Emergency Medical Service
IF: Implementation facilitation
IRB: Institutional Review Board
LC: Local champion
MOUD: Medication for opioid use disorder
NIDA: National Institute on Drug Abuse
NH: New Hampshire
NY: New York
ORCA: Organizational readiness to change assessment
OTP: Opioid Treatment Program
OUD: Opioid use disorder
PARiHS: Promoting Action on Research in Health Services
PDMP: Prescription Drug Monitoring Program
PhenX: Phenotypes and eXposures
UDS: Urine drug screen
SL-BUP: Sublingual buprenorphine
TLFB: Timeline follow-back
XR-BUP: Extended-release buprenorphine
